# Giant Basal Cell Carcinoma of the Scalp

**Published:** 2016-06-28

**Authors:** Aastha Tandon, Paul J. Therattil, Edward S. Lee, Ravi J. Chokshi

**Affiliations:** ^a^Divisions of Surgical Oncology, Department of Surgery, Rutgers New Jersey Medical School, Newark; ^b^Plastic and Reconstructive Surgery, Department of Surgery, Rutgers New Jersey Medical School, Newark

**Keywords:** basal cell carcinoma, metastasis, metastatic workup, scalp defect, scalp reconstruction

**Figure:**
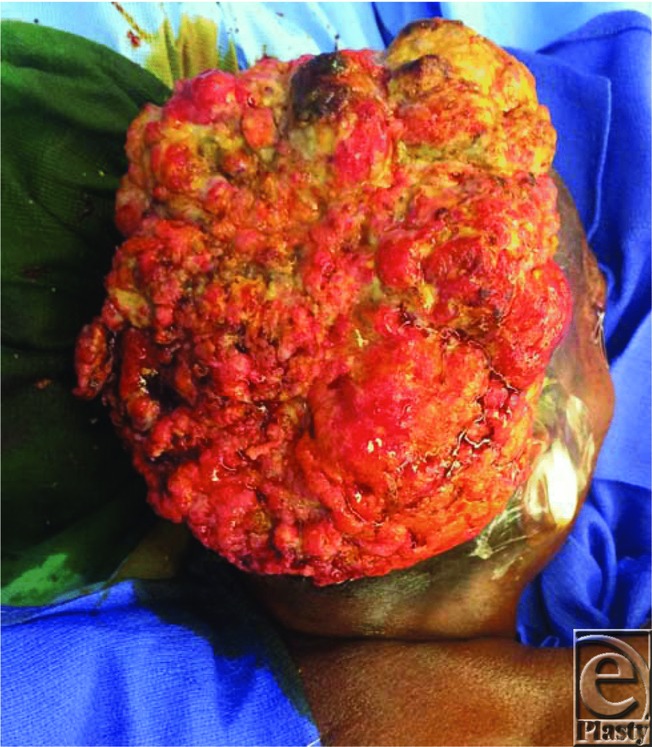


## DESCRIPTION

A 60-year-old woman presented with a 15 × 15-cm fungating tumor of the posterior scalp.

## QUESTIONS

1. **Can basal cell carcinoma (BCC) metastasize?**
2. **What diagnostic imaging should be used preoperatively, if any?**
3. **What is the role of sentinel lymph node biopsy in BCC?**
4. **What are the reconstructive options for a defect of this size at the scalp?**

## DISCUSSION

Basal cell carcinomas tend to be slow-growing and painless tumors, which may allow patients to ignore signs and defer immediate treatment. The result of a neglected BCC can be large fungating masses that have an increased risk of metastasis. Adequate preoperative evaluation of such lesions includes tissue biopsy, radiologic studies, and a metastatic workup in some cases. Metastatic BCC is extremely rare, with only a few hundred cases described in the literature. The incidence may be as small as 0.0028% to 0.1%.^1^ Metastatic workup should be considered for lesions at higher risk for developing distant disease, which includes neglected lesions, lesions larger than 3 cm, recurrent lesions, lesions with perineural or vascular invasion, lesions with neuroendocrine differentiation, and lesions of the scalp and ear.^1^

It is critical to evaluate for metastases, as a distant metastatic BCC has a much poorer prognosis (median survival of 10–14 months).^2^ Disease most often spreads to the lung, bone, and skin. Metastasis to the lymph nodes has a slightly better prognosis, with median survival of 3.6 years.^3^ It is important to recognize the potential for poor prognosis as, in addition to treatment options, it may affect the reconstruction options pursued. For late stages of BCC, computed tomography and magnetic resonance imaging are the primary imaging modalities. Although the indications for imaging in advanced BCCs are still evolving, generally stage III and IV disease (bony or perineural involvement) require imaging.^4^ Computed tomography may be more sensitive in demonstrating visceral disease and bone involvement, whereas magnetic resonance imaging is a better option to detect local disease, orbital involvement, perineural disease, and brain metastasis or invasion.^4^ Positron emission tomography/computed tomography is indicated to detect distant metastatic disease and evaluate response to treatment.

Sentinel lymph node biopsy (SLNB) is a minimally invasive technique using adjacent intradermal injection of radiotracer to detect the draining nodes with lymphoscintigraphy. In treatment of melanoma, SLNB is used to determine whether regional lymphadenectomy is needed, as well as for staging, prognostication, and patient selection for adjuvant treatment. While other select nonmelanoma skin cancers have clear indications for SLNB (Merkel cell carcinoma, sweat gland carcinoma, high-risk squamous cell carcinoma), the indications for SLNB in metastatic BCCs are less clear.^1^ There are several reasons for this uncertainty, including lack of association between lymphadenectomy and disease cure in BCCs and because the rate of metastatic BCCs is so low that the risks of undergoing the procedure may outweigh the benefit. Sentinel lymph node biopsy should be considered at the surgeon's discretion, especially when there is palpable lymphadenopathy or when lesions demonstrate high-risk features such as lymphatic invasion.^1^^,^^4^

**Figure 1:**
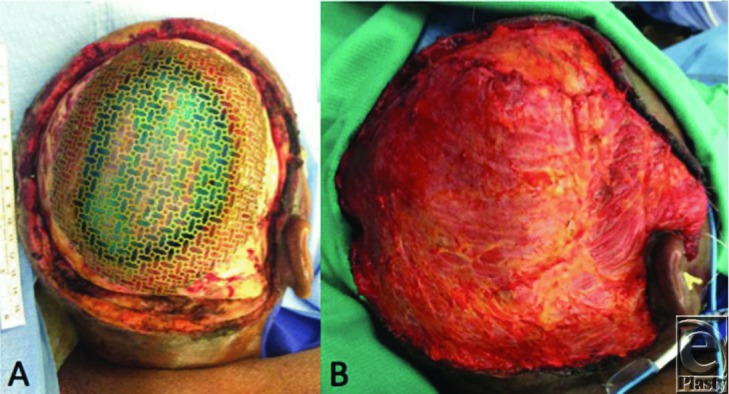
Intraoperative view of (a) near-total scalp defect after resection of giant basal cell carcinoma and mesh cranioplasty, and (b) reconstruction with free latissimus dorsi flap.

**Figure 2:**
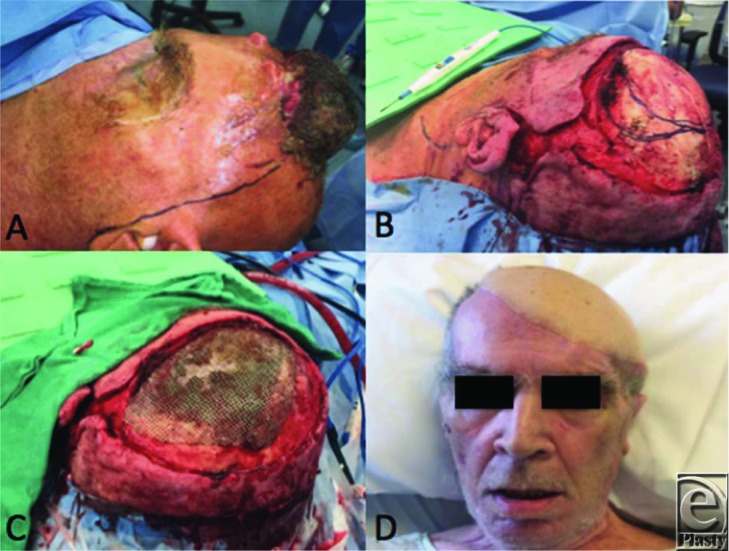
A 61-year-old man presented with a (a) 30 × 30-cm fungating giant basal cell carcinoma of the scalp that was (b) excised and reconstructed with (c) a mesh cranioplasty and (d) free anterolateral thigh flap.

Reconstruction of large scalp defects may be treated with skin grafting, local flaps, locoregional flaps, or free flaps depending on the extent of the defect and the depth of resection. Defects with periosteum intact at the base may be treated with skin grafting alone. Even if periosteum has been resected, the outer table of the skull may be burred and then grafted. Skin grafts, however, have a poor aesthetic result with alopecia and poor color match and should be considered if the patient cannot tolerate more extensive procedures. If the patient can tolerate a longer procedure, then flap reconstruction is indicated along with potential reconstruction of the skull itself if full-thickness bone has been resected. Unlike other regions of head and neck, local flaps are limited to smaller defects (2–25 cm^2^) by the scalp's inelasticity and thus large rotational flaps or multiple scalp flaps may be needed. Despite this, well-constructed flap combinations may cover defects up to 50% of the total area of the scalp with skin grafting of the donor sites. Tissue expansion prior to reconstruction may also be helpful in gaining donor site area. Regional flaps (latissimus dorsi, pectoralis major, temporoparietal fascia) are possible but limited in their arc of rotation and thus limited in their usefulness. Defects reaching 100 to 200 cm^2^ typically require free tissue transfer.^5^^,^^6^ For large defects, the latissimus dorsi muscle or the anterolateral thigh free flap can reliably provide a large area of coverage. Figure 1 demonstrates a patient who underwent reconstruction with a free latissimus dorsi flap reconstruction of a large scalp defect after giant BCC resection, whereas Figure 2 demonstrates a patient with free anterolateral thigh flap reconstruction for tumor resection.
